# The C-Terminal Transmembrane Domain of Cowpea Mild Mottle Virus TGBp2 Is Critical for Plasmodesmata Localization and for Its Interaction With TGBp1 and TGBp3

**DOI:** 10.3389/fmicb.2022.860695

**Published:** 2022-04-15

**Authors:** Chong Jiang, Shiqi Shan, Yue Huang, Chenyang Mao, Hehong Zhang, Yanjun Li, Jianping Chen, Zhongyan Wei, Zongtao Sun

**Affiliations:** State Key Laboratory for Managing Biotic and Chemical Threats to the Quality and Safety of Agro-Products, Key Laboratory of Biotechnology in Plant Protection of MOA of China and Zhejiang Province, Institute of Plant Virology, Ningbo University, Ningbo, China

**Keywords:** cowpea mild mottle virus, triple gene block proteins, intercellular movement, plasmodesmata targeting, transmembrane domain

## Abstract

The movement of some plant RNA viruses is mediated by triple gene block (TGB) proteins, which cooperate to transfer the viral genome from cell to cell through plasmodesmata. Here, we investigated the function of the TGB proteins of cowpea mild mottle virus (CPMMV; genus *Carlavirus*, family *Betaflexiviridae*), which causes severe damage to soybean production. Subcellular localization experiments demonstrated that TGBp1 and TGBp3 were localized to the endoplasmic reticulum (ER), plasmodesmata (PD) and nucleus in *Nicotiana benthamiana* leaves. TGBp2 was unusually localized to PD. In protein interaction assays TGBp2 significantly enhanced the interaction between TGBp3 and TGBp1. Interaction assays using deletion mutants showed that the C-terminal transmembrane (TM) domain of TGBp2 is critical for its localization to PD and for its interaction with TGBp1 and TGBp3.

## Introduction

Plant viruses move from cell to cell via plasmodesmata (PD) and so spread systemically in infected plants. This movement requires the assistance of virus-encoded specialized movement proteins (MPs), which target the PD and increase their size exclusion limit (SEL) so as to enable the RNA or an RNA-MP complex (ribonucleoprotein complex, RNP) to pass through the PD and spread to adjacent cells (Heinlein, 2015). Studies on the 30-kDa protein of tobacco mosaic virus (TMV) were the first to suggest that the intercellular spread of a plant virus can be mediated by a single viral protein (Leonard and Zaitlin, 1982). Some plant RNA viruses do not have gene products similar to the TMV MP, but instead encode three proteins in partially overlapping open reading frames (ORFs), named a triple gene block (TGB). Based on the positions of TGB genes in the block, the TGB-encoded proteins are called TGBp1, TGBp2, and TGBp3 (Skryabin et al., 1988). Plant viruses that encode TGB movement proteins are classified in the families *Alphaflexiviridae* (including the genera *Potexvirus, Allexivirus*, and *Mandarivirus*), *Betaflexiviridae* (including the genera *Carlavirus* and *Foveavirus*), *Virgaviridae* (including the genera *Hordeivirus, Pomoviru*s, and *Pecluvirus*) and *Benyviridae* (including the genus *Benyvirus*) (Solovyev et al., 2012).

In recent years, the structures and functions of the three TGB proteins have been extensively characterized (Verchot-Lubicz et al., 2010). Two major classes of TGB proteins, potex- and hordei-like TGB proteins, have been recognized based on substantial differences in their TGBp1 and TGBp3s, while the TGBp2 is highly conserved throughout both groups (Solovyev et al., 2012). Potex-like TGBp1s are 24–26 kDa and contain a helicase-like (HELD) domain, which is essential for RNA binding and helicase activity (Lin et al., 2004; Han et al., 2007). TGBp1s of the hordei-like group are larger (39–63 kDa) and have an N-terminal RNA-binding domain in addition to the HELD domain (Kalinina et al., 2001, 2002; Makarov et al., 2009). TGBp1 can increase the SEL of PD and promote the translation of viral RNA, whereas TGBp2 is an integral membrane protein that possesses two transmembrane (TM) domains, and it is associated with the endoplasmic reticulum (ER) and ER-derived granular vesicles (Morozov and Solovyev, 2003; Howard et al., 2004). TGBp2 integrates into the ER membranes in a U-like structure with the conserved hydrophilic central region within the ER lumen, and both termini exposed to the cytosol (Morozov and Solovyev, 2003). Deleting either of the TM domains of the TGBp2 protein or replacement of two conserved Cys residues at the C-terminus inhibited viral cell-to-cell movement and systemic infection to a great extent (Ju et al., 2007). TGBp3 proteins contain a single TM domain at the N-terminus and the C-terminal region is exposed to the cytosol. In addition, there is a sorting signal at the C-terminus of TGBp3, which can target TGBp2/TGBp3 vesicles or complexes to the cortical ER tubules near PD and shows PD localization (Lim et al., 2008; Lee et al., 2010; Wu et al., 2011; Chou et al., 2013).

The three TGB proteins work together to deliver the viral genome through the PD and complete viral transport in infected plants. TGBp2 co-localizes with TGBp3 in the ER or TGBp2-induced ER-derived vesicles and forms a TGBp2/TGBp3 complex (Morozov and Solovyev, 2003; Verchot-Lubicz et al., 2010) which is targeted to PD through the sorting signals of TGBp3 (Schepetilnikov et al., 2005; Samuels et al., 2007). Additionally, a ribonucleoprotein complex containing viral RNA and TGBp1, interacts via the TGBp1 with the TGBp2-TGBp3 complex and assists the viral proteins in passing through PD (Erhardt et al., 2000; Zamyatnin et al., 2004; Lim et al., 2008). It is also known that either TGBp2 or TGBp3 alone can enhance the PD localization of TGBp1, and that the C-terminal regions of both TGBp1 and TGBp2 play an important role in strengthening the TGBp2- and TGBp3-dependent PD location of TGBp1 (Verchot-Lubicz et al., 2010).

Cowpea mild mottle virus (CPMMV) is a positive-sense single-stranded RNA virus belonging to the genus *Carlavirus* (Almeida et al., 2003; Zanardo et al., 2014). It was first reported to infect cowpea in Ghana but was then found to infect legumes and other crops across the world (Brunt and Kenten, 1973). Recent studies have shown that CPMMV is also widely distributed in China, causing severe damage to soybean yields (Wei et al., 2021). The CPMMV genome has six ORFs, which encode an RNA-dependent RNA polymerase protein (RdRp), triple gene block proteins (TGBp1, p2, and p3), coat protein (CP), and a cysteine-rich protein with nucleic acid binding activity (CRP) (Menzel et al., 2010). Although the molecular mechanisms and functions of the three TGB proteins have been well characterized in many plant RNA viruses, their function in members of the genus *Carlavirus* has not been studied.

In this study, the TGB genes of the CPMMV were cloned, and the subcellular localization of their proteins in *Nicotiana benthamiana* were investigated. It was found that the TGBp2 of CPMMV displayed unique PD localization and strongly interacted with TGBp1 and TGBp3. Meanwhile, BiFC and Co-immunoprecipitation assays showed that TGBp1 exhibited weak interactions with TGBp3 in the cytoplasm, and that TGBp2 could significantly enhance the interaction between TGBp1 and TGBp3, and form a protein complex appearing at or near the cell wall. Furthermore, the C-terminal TM domain of TGBp2 was shown to play an important role in its PD localization and in its interaction with TGBp1 and TGBp3.

## Materials and Methods

### Total RNA Extraction and Triple Gene Block Cloning

Soybean leaves infected with the CPMMV isolate CPMMV_AH_SZ (Acc. no. MN908944) were collected, and total RNA was extracted using TRIzol reagent (Invitrogen, United States) according to the manufacturer’s protocols. Subsequently, about 1.5 μg of total RNA was used to synthesize cDNA using a reverse transcription kit (Tiangen Company, China). In addition, full-length *TGBp1, TGBp2* and *TGBp3* were amplified from the cDNA using KOD FX polymerase (Toyobo, Japan), and cloned into the pCAMBIA and pBIN binary vectors. The primers employed are listed in Supplementary Table 2.

### Sequence Analysis of TGBp2 and Truncated Mutant Construction

The transmembrane domains of the TGBp2 protein were predicted using several methods: HMMTOP^1^ (Tusnady and Simon, 2001), DAS^2^ (Cserzö et al., 1997) and SPLIT^3^ (Juretić et al., 1993). Truncated mutants of TGBp2 were constructed according to the predicted domains, and the amino acid residues of TGBp2 (1 aa-80 aa and 29 aa-106 aa) were cloned into the pCAMBIA1305-GFP binary vector to generate pCAMBIA1305-TGBp2-ΔC26-GFP and pCAMBIA1305-TGBp2-ΔN28-GFP, respectively. The primers used are shown in Supplementary Table 2.

### Subcellular Localization Assays

For subcellular localization in *N. benthamiana*. GFP was fused to either the N-terminus or the C-terminus of TGBp1, TGBp2, and TGBp3 under the control of CaMV 35S. Full-length *TGBp1*, *TGBp2*, and *TGBp3* were cloned into the pCAMBIA1305-GFP binary vector to generate pCAMBIA1305-TGBp1-GFP, pCAMBIA1305-TGBp2-GFP, pCAMBIA1305-TGBp3-GFP, and cloned into the pBIN-GFP binary vector to generate pBIN-GFP-TGBp1, pBIN-GFP-TGBp2, and pBIN-GFP-TGBp3, while the recombinant binary vectors were separately introduced into *Agrobacterium tumefaciens* strain GV3101 by electroporation and cultured at 28°C for 2 days. Then, the bacterial cultures were collected and suspended in the infiltration solution [10 mM MgCl_2_, 10 mM MES (pH 5.6), 200 μM Acetosyringone] at an appropriate concentration (OD_600_ = 1.0∼1.5). After about 2 h, the *A. tumefaciens* solutions were infiltrated into 4–6 weeks-old seedlings of *N. benthamiana*.

Confocal imaging was performed using a laser scanning confocal microscope (Nikon AIR, Japan). GFP fluorescence was excited at 488 nm and emission was captured at 500–530 nm, RFP fluorescence was excited at 568 nm and captured at 580–630 nm, while YFP fluorescence was excited at 514 nm and captured at 525–650 nm.

### Bimolecular Fluorescence Complementation Assays

The full-length *TGBp1*, *TGBp2*, and *TGBp3* and truncated mutants *TGBp2-*Δ*C26* and *TGBp2-*Δ*N28* were cloned into pCAMBIA1305-cYFP and pCAMBIA1305-nYFP vectors. The recombinant binary vectors were transformed into *Agrobacterium tumefaciens* strain GV3101 by electroporation. 6-week-old *N. benthamiana* seedlings were used for agroinfiltration experiments. After 48 h of infiltration, the YFP fluorescence signal was captured using the confocal laser scanning microscope (Nikon AIR, Japan). For quantify the yellow fluorescence produced by TGB1-cYFP and TGB3-nYFP in BiFC assay, the mean intensity of the fluorescence in whole confocal images were measured using ImageJ software (version: 1.53a), more than five images from at least three independent experiments were measured for each analysis.

### Co-immunoprecipitation Assays

For Co-IP assays, the full-length TGB proteins were expressed separately from pCAMBIA1305-Flag and pCAMBIA1305-Myc binary vectors. The following combinations were infiltrated into 6-week-old *N. benthamiana*: TGBp1-Myc and TGBp2-Flag/GFP-Flag, TGBp3-Myc and TGBp2-Flag/GFP-Flag, TGBp1-Myc and TGBp3-Flag, TGBp1-Myc and TGBp2-GFP and TGBp3-Flag. After 36 h of infiltration, the leaves were collected and ground into powder with liquid nitrogen. Total protein was extracted from 0.5 g samples with 1 ml pre-chilled IP lysis buffer (Thermo Fisher Scientific, United States), and 10 mM DTT, a protease inhibitor (Roche, Switzerland), which was then incubated on ice for 10 min and centrifuged at 1,000 × *g* for 20 min at 4°C. The supernatant was then carefully transferred to a new microcentrifuge tube (100 μl as input sample), 5 μL Myc-Trap Magnetic Agarose (Cat No. Ytma, Proteintech) or MonoRab™ Anti-DYKDDDDK Magnetic Beads (Cat No. L00835-1, GenScript) pre-washed with 1x PBS were added, and incubated at 4°C for 2 h. After that, the supernatant was removed and the magnetic beads were washed at least three times in cold 1x PBS buffer with protease inhibitor cocktail to remove non-specific binding proteins. Finally, the corresponding volume of 5 × SDS-PAGE loading buffer was added and boiled for 5 min before immunoblot analysis.

## Results

### Subcellular Localization of the Triple Gene Block Proteins in *N. benthamiana*

**FIGURE 1:**
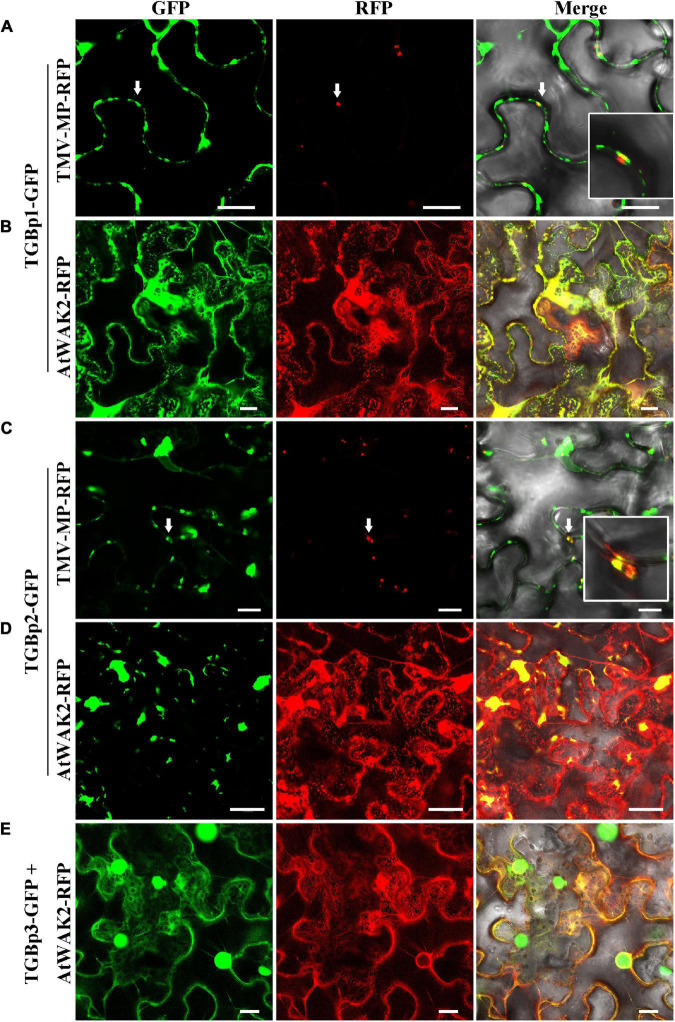
Subcellular localization analysis of CPMMV TGB-GFP proteins in *N. benthamiana* leaf cells. Images of *N. benthamiana* leaf cells co-expressing PD marker TMV-MP-RFP with TGBp1-GFP **(A)** and TGBp2-GFP **(C)** and of those expressing the ER marker AtWAK2-RFP with TGBp1-GFP **(B)**, TGBp2-GFP **(D)** and TGBp3-GFP **(E)**. The co-localized site is indicated by the white arrow and is magnified in the Merge panel on the right. Confocal images were taken at 48 h post-infiltration. Scale bars = 20 μm.

**FIGURE 2:**
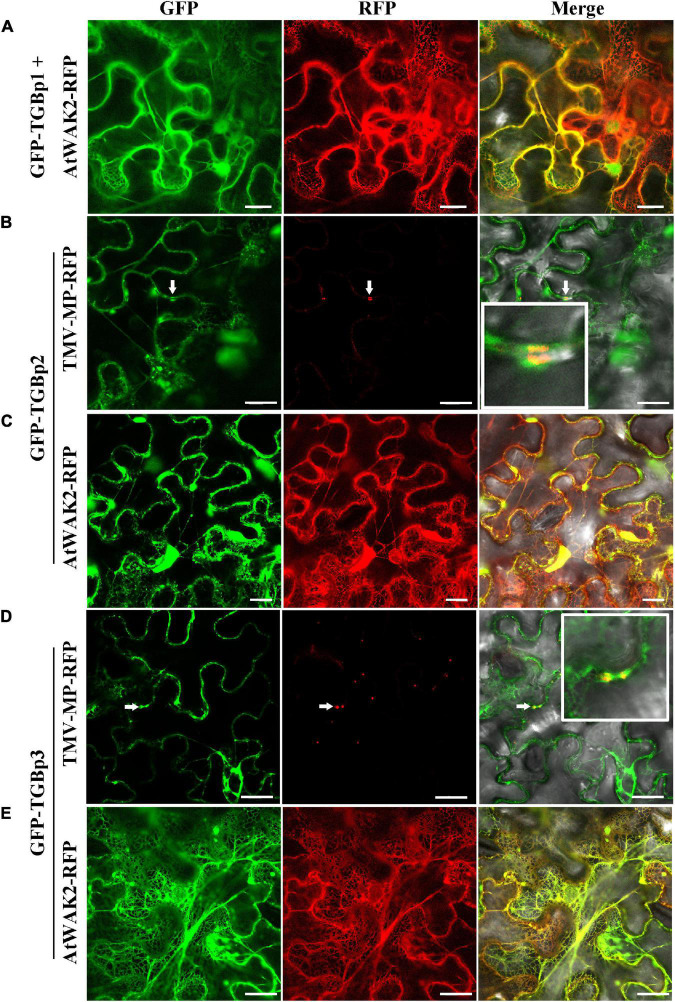
Subcellular localization analysis of CPMMV GFP-TGB proteins in *N. benthamiana* leaf cells. Images of *N. benthamiana* leaf cells co-expressing ER marker AtWAK2-RFP with GFP-TGBp1 **(A)**, GFP-TGBp2 **(C)** and GFP-TGBp3 **(E)** and of those expressing the PD marker TMV-MP-RFP co- with GFP-TGBp2 **(B)** and GFP-TGBp3 **(D)**. The co-localized site is indicated at the white arrow and is magnified in the Merge panel on the right. Confocal images were taken at 48 h post-infiltration. Scale bars = 20 μm.

To study the subcellular localization of each of the TGB proteins, we made two GFP fusions for each of the TGBps to express GFP fused to either their N- or C-terminus (GFP-TGBs and TGBs-GFP, respectively). Free GFP, used as a control, was targeted to the cytoplasm and to the nucleus (Supplementary Figure 1G). The subcellular distribution of TGBp1-GFP differed from that of GFP-TGBp1. TGBp1-GFP predominantly targeted the cytoplasm and the periphery of the cell, while GFP-TGBp1 targeted the nucleoplasm but not the nucleolus, and network-like structures in the cytoplasm (Supplementary Figures 1A,B). To examine the localization of TGBp1 in more detail, TGBp1-GFP or GFP-TGBp1 were co-expressed with RFP fluorescent marker proteins for the ER (AtWAK2-RFP) (Gomord et al., 1997; He et al., 1999) or PD (TMV-MP-RFP) (Nick, 2008; Rodrigues et al., 2015). TGBp1-GFP formed paired punctate fluorescent foci at the PD of adjacent cells, and largely overlapped with the PD marker TMV-MP-RFP (Figure 1A). TGBp1-GFP and AtWAK2-RFP also overlapped to provide a yellow fluorescence signal as a network-like structure, indicating that TGBp1-GFP is also localized partly at the ER (Figure 1B). A similar pattern of ER localization was observed in the combination of GFP-TGBp1 and AtWAK2-RFP (Figure 2A). N-terminally tagged GFP-TGBp1 showed nuclear localization (Figure 2A), but not the PD localization of the C-terminally tagged TGBp1-GFP (Figure 1A), which is consistent with results reported for other viral TGBp1 proteins (Samuels et al., 2007; Rebelo et al., 2008). These results indicate that TGBp1 is mainly localized to the ER, PD and nucleus of *N. benthamiana* leaf cells, and that different fusion positions (GFP fused at the N- or C-terminus) have an impact on its localization.

In studies of the subcellular localization of TGBp2, GFP fluorescence was concentrated at or near the cell wall irrespective of the terminus to which GFP was fused (Supplementary Figures 1C,D). To determine the nature of these punctate compartments, TGBp2-GFP or GFP-TGBp2 were then co-expressed with the ER and PD RFP marker proteins as described above. Both TGBp2-GFP and GFP-TGBp2 co-localized substantially with both the PD marker TMV-MP-RFP (Figures 1C, 2B) and AtWAK2-RFP-tagged ER structures (Figures 1D, 2C). The results are significant because TGBp2 proteins are not usually localized to PD (Table 1), with the exception of BaMV (genus *Potexvirus*) which has little sequence similarity to that of CPMMV (28% aa identity).

**TABLE 1:** Subcellular localization of TGB proteins in some plant RNA viruses.

TGBs	Cytoplasm	Nucleus	PD	ER	References
PVX-TGBp1	**√**		**√**		Samuels et al., 2007
BaMV-TGBp1	**√**	**√**			Chou et al., 2013
PMTV-TGBp1	**√**	**√**			Zamyatnin et al., 2004
BSMV-TGBp1	**√**				Lim et al., 2009
CPMMV-TGBp1	**√**	**√**	**√**	**√**	This study
GRSPaV-TGBp1	**√**	**√**			Rebelo et al., 2008
PSLV-TGBp1	**√**	**√**			Semashko et al., 2012
PVX-TGBp2				**√**	Samuels et al., 2007
BaMV-TGBp2			**√**		Chou et al., 2013
PMTV-TGBp2				**√**	Haupt et al., 2005
BSMV-TGBp2				**√**	Lim et al., 2009
CPMMV-TGBp2			**√**	**√**	This study
GRSPaV-TGBp2				**√**	Rebelo et al., 2008
PSLV-TGBp2				**√**	Solovyev et al., 2000
PVX-TGBp3				**√**	Samuels et al., 2007
BaMV-TGBp3			**√**	**√**	Wu et al., 2011
PMTV-TGBp3			**√**	**√**	Haupt et al., 2005
BSMV-TGBp3			**√**		Lim et al., 2009
CPMMV-TGBp3	**√**	**√**	**√**	**√**	This study
GRSPaV-TGBp3				**√**	Rebelo et al., 2008
PSLV-TGBp3				**√**	Solovyev et al., 2000

Experiments with the two GFP fusions of TGBp3 showed that the C-terminal GFP-tagged TGBp3-GFP targeted the cytoplasm and the nucleus. An enlarged view shows that TGBp3-GFP was distinct from the ER (Figure 1E) and distributed throughout the nucleus excluding the nucleolus (Supplementary Figure 1E). In contrast, expression of the N-terminal GFP-tagged GFP-TGBp3 resulted in a network-like structure with punctate fluorescent foci at or near the cell wall (Supplementary Figure 1F). Co-expression with the RFP markers confirmed the ER localization of GFP-TGBp3 (Figure 2E) while the punctate fluorescent foci of GFP-TGBp3 largely overlapped with the PD marker TMV-MP-RFP (Figure 2D).

### Interactions Between the Triple Gene Block Proteins

**FIGURE 3:**
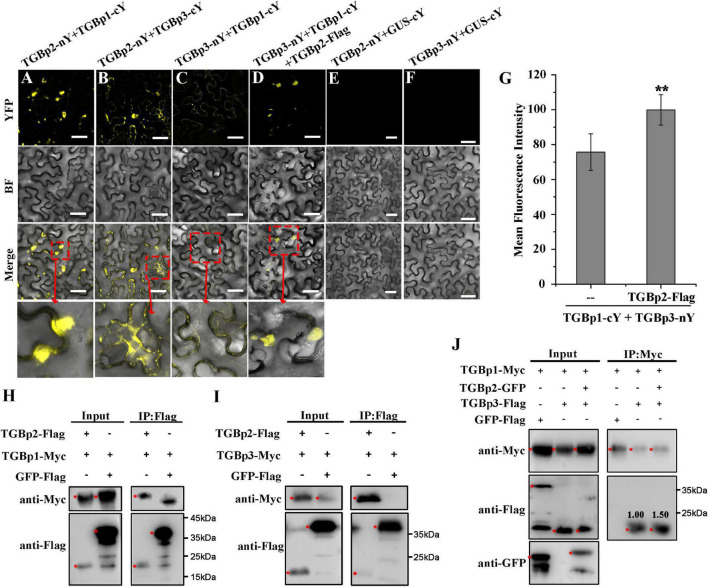
Analysis of interactions between CPMMV TGB proteins *in vivo*. **(A–F)** BiFC assays to detect physical interactions between TGBp2-nY and TGBp1-Cy **(A)**, TGBp2-nY and TGBp3-cY **(B)**, TGBp3-nY and TGBp1-cY **(C)**, TGBp3-nY, TGBp1-Cy, and TGBp2-Flag **(D)**. Agro-infiltration with TGBp2-nY + GUS-cY **(E)** and TGBp3-nY + GUS-cY vector **(F)** served as negative controls. The areas marked with red boxes in the Merge row are enlarged below. Scale bars = 50 μm. Mean fluorescence intensities of the combination of TGBp3-nY and TGBp1-cY in the presence and absence of TGBp2-Flag **(G)**. Data were from at least three independent experiments. Values are means ± SD, *n* ≥ 5. **P < 0.01, Student’s *t*-test. **(H–J)** Interactions between CPMMV TGB proteins detected by the Co-IP assays. TGBp1 **(H)** and TGBp3 **(I)** were co-immunoprecipitated by TGBp2-Flag using Flag beads in a total leaf extract of *N. benthamiana*. The co-immunoprecipitated proteins were detected by anti-Myc antibody. Co-IP assays **(J)** confirmed that TGBp2 increases the interaction between TGBp1 and TGBp3. The co-immunoprecipitated proteins were detected by anti-Flag antibody. The red asterisks indicating bands of TGBps fusion protein and GFP-Flag in western blots. Relative protein amounts shown were determined using ImageJ software.

Because interactions between the TGB proteins are of great importance for the cell-to-cell movement of plant viruses (Verchot-Lubicz et al., 2010), we next used BiFC assays to investigate if and how the three TGB proteins of CPMMV cooperate to perform their functions. Co-expression of TGBp2-nYFP with either TGBp1-cYFP or TGBp3-cYFP produced strong fluorescence signals in *N. benthamiana* leaves, but there were no detectable signals in the negative control groups (Figures 3A,B). These results demonstrate that TGBp2 interacts with both TGBp1 and TGBp3 *in vivo*. Co-immunoprecipitation (Co-IP) assays also demonstrated interactions between TGBp2 fusion protein and either TGBp1 (Figure 3H) or TGBp3 (Figure 3I) but not with the negative control GFP-flag. Taken together, these results suggest that TGBp2 interacts with both TGBp1 and TGBp3.

When the relationship between TGBp1 and TGBp3 was investigated by BiFC assays, a weaker reconstituted YFP signal was observed in *N. benthamiana* leaves co-expressing TGBp1-cYFP and TGBp3-nYFP (Figure 3C). Since TGBp2 interacts with both TGBp1 and TGBp3 (see above), the role of TGBp2 in the TGBp1-TGBp3 interaction was then tested by BiFC and Co-IP assays. In the presence of TGBp2-Flag, the YFP fluorescence signal produced by co-expression of TGBp1-cYFP and TGBp3-nYFP was enhanced to a great extent and appeared at or near the cell wall (Figure 3D). Analysis of the fluorescence intensity by ImageJ (version 1.53a) confirmed that co-expression of TGBp2-Flag significantly increased the fluorescence intensity of TGBp1-nY + TGBp3-cY (from 75.7 ± 10.44 to 95.9 ± 8.72) (Figure 3G). Further Co-IP results also showed that TGBp3-Flag was co-immunoprecipitated by TGBp1-Myc, and the ability of TGBp1-Myc and TGBp3-Flag to associate was markedly enhanced in the presence of TGBp2 protein (Figure 3J). In addition, RT-qPCR (Supplementary Figure 2A) and western-blotting (Supplementary Figure 2B) results showed that there was no significant difference in the expression of TGBp1-cYFP and TGBp3-nYFP in the presence or absence of TGBp2. Collectively, these results suggest that TGBp2 directly enhances the interaction between TGBp1 and TGBp3, and forms a complex with them.

### The C-Terminal Transmembrane Domain of TGBp2 Is Essential for Plasmodesmata Localization

**FIGURE 4:**
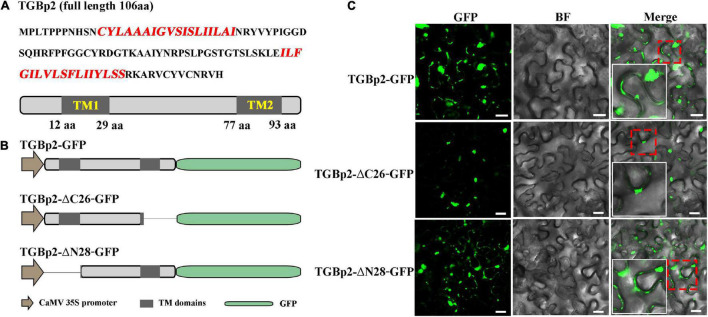
Effects of TGBp2 transmembrane domains on its localization in *N. benthamiana* leaf cells. **(A)** The amino acid sequence of CPMMV TGBp2, with the sequence of the transmembrane (TM) domains highlighted in red. The genome organization is shown below the sequence, with TM domains represented as gray boxes. The location of the TM domains was predicted by HMMTOP. **(B)** Schematic representation of the TGBp2 truncated mutants used in the BiFC assay. The N-terminal (TGBp2-ΔN28) and C-terminal truncated mutants (TGBp2-ΔC26) were constructed according to the HMMTOP model prediction results. GFP was fused to the C terminal of truncated mutants under the control of CaMV 35S promoter. **(C)** Subcellular localization analysis of wild-type TGBp2-GFP protein, and the truncated mutants TGBp2-ΔC26-GFP and TGBp2-ΔN28-GFP in *N. benthamiana* leaves. Selected regions in red boxes are magnified in the insets. Scale bars = 20 μm.

Predictions of the protein motifs of CPMMV TGBp2 using HMMTOP, DAS, and SPLIT (Supplementary Table 1), revealed that TGBp2 has two transmembrane (TM) domains, one near the N-terminus and the other near the C-terminus (Figure 4A). To investigate whether these TM domains are essential for the targeting of TGBp2 to PD, truncated mutants were constructed and used in localization experiments. When the C-terminal 26 amino acids were truncated (TGBp2-ΔC26), the PD localization of TGBp2 was abolished and only aggregated fluorophores appeared near the cell wall (Figures 4B,C). In contrast, when the N-terminal 28 amino acids were truncated (TGBp2-ΔN28) the TGBp2 protein localization pattern was similar to that of the wild type (Figures 4B,C). Therefore, these results suggest that the TM domain at the C-terminus, but not that at the N-terminus, is essential for TGBp2 to target PD.

### The C-Terminal Transmembrane Domain of TGBp2 Is Required for the TGBp1 Interaction

**FIGURE 5:**
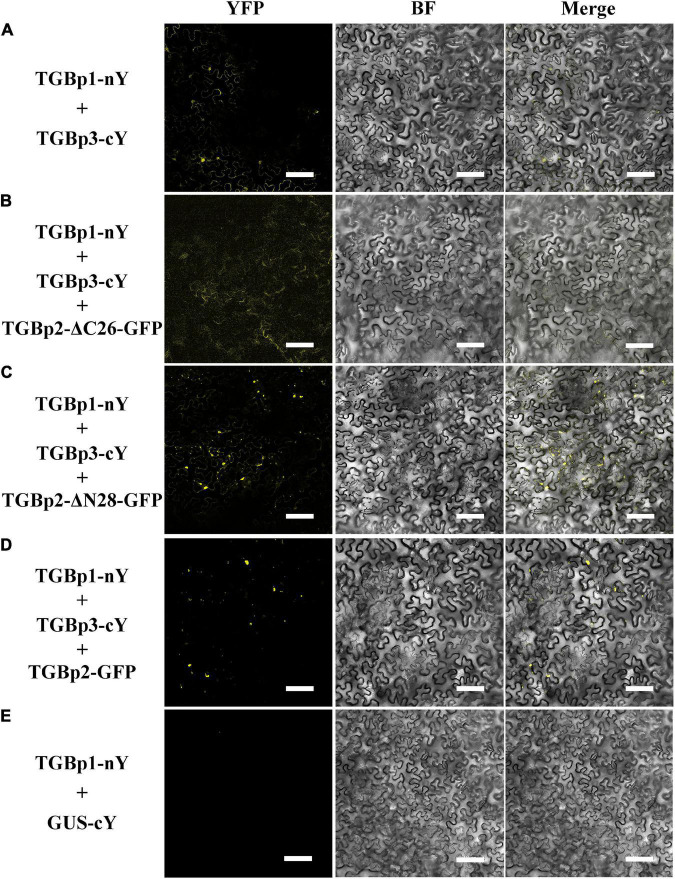
Effects of the TGBp2 TM domains on the interaction between TGBp1 and TGBp3 *in vivo.* BiFC assays were used to detect physical interactions between TGBp1-nY and TGBp3-cY **(A)**, TGBp1-nY, TGBp3-cY, and TGBp2-ΔC26-GFP **(B)**, TGBp1-nY, TGBp3-cY, and TGBp2-ΔN28-GFP **(C)**, and TGBp1-nY, TGBp3-cY, and TGBp2-GFP as a positive control **(D)**. TGBp3-nY and GUS-cY vector **(E)** served as a negative control (Scale bars = 100 μm).

Since only the C-terminal TM domain of TGBp2 affects its localization, we wondered whether either of the TM domains affect the interaction between TGBp2 and the other TGB proteins. Truncated mutants of TGBp2 were first co-expressed with TGBp1-nYFP and TGBp3-cYFP in *N. benthamiana* leaves. The wild-type TGBp2 enhanced the interaction between TGBp1-cYFP and TGBp3-nYFP giving stronger reconstituted YFP signals (Figures 5A,D,E), but there was no such enhancement when the TGBp2 C-terminal TM domain was truncated (Figure 5B). The N-terminal mutant TGBp2-ΔN28-GFP enhanced the fluorescence like the wild type TGBp2 (Figures 5C,D). Thus, the C-terminal of TGBp2 is critical for promoting the interaction between TGBp1 and TGBp3.

**FIGURE 6:**
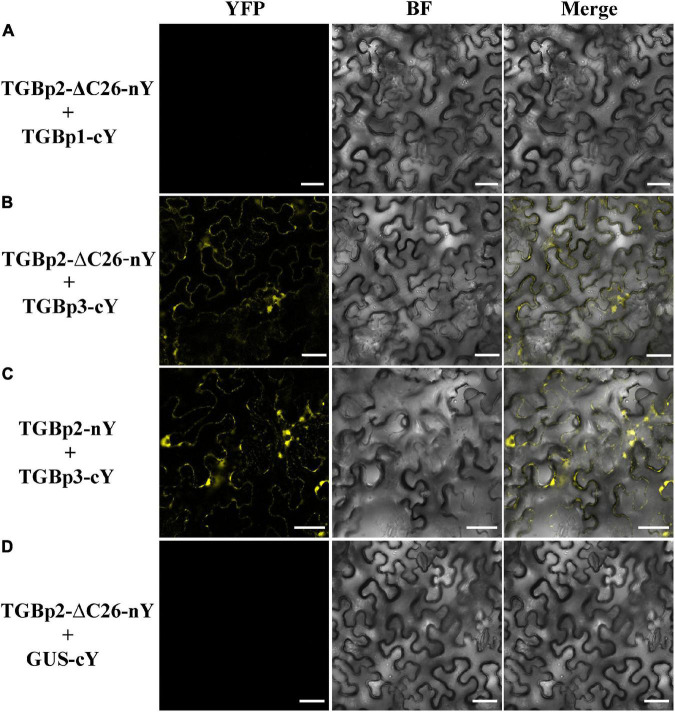
Analysis of interactions between the TGBp2 C-terminal truncation mutant and TGB1 or TGBp3 *in vivo.* BiFC assays were used to detect physical interactions between TGBp2-ΔC26-nY and TGBp1-cY **(A)**, TGBp2-ΔC26-nY and TGBp3-cY **(B)**, and TGBp2-nY and TGBp3-cY as a positive control **(C)**. TGBp2-ΔC26-nY + GUS-cY vector **(D)** served as a negative control (Scale bars = 50 μm).

Since TGBp2 enhances the interaction between TGBp1 and TGBp3 by forming a protein complex with TGBp1 and TGBp3 (Figure 3D), we then used BiFC assays to test whether the C-terminal TM domain of TGBp2 was directly involved in the interaction between TGBp2 and the other TGB proteins. Reconstituted YFP signals at or near the cell wall, indicating a positive interaction, occurred when full length TGBp2-nYFP and TGBp3-cYFP were co-infiltrated (positive control, Figure 6C). The truncated mutant TGBp2-ΔC26-nYFP interacted with TGBp3-cYFP (Figure 6B) but not with TGBp1-cYFP (Figure 6A) and negative control GUS-cYFP (Figure 6D). The results indicate that that the C-terminal TM domain of TGBp2 is vital for its interaction with TGBp1 and that this interaction is also significant for the role of TGBp2 in enhancing the interaction between TGBp1 and TGBp3.

## Discussion

Unlike viruses that encode only a single movement protein, those viruses that encode TGB proteins require those proteins to cooperate with each other to assist the viral genome to target PD and infect adjacent host cells. CPMMV is a destructive pathogen of soybean, but the interaction and subcellular localization of its TGBs have not been reported. The results reported here will provide a better understanding of how CPMMV spreads in infected plants.

Previous studies have demonstrated that the TGBp1 targets the cytoplasm and the nucleus when expressed alone and that its transport to PD generally requires the assistance of TGBp2 and TGBp3 (Erhardt et al., 2000). The TGBp2- and TGBp3-dependent PD localization of TGBp1 has been reported for many plant viruses, including potato mop-top virus (PMTV) (Zamyatnin et al., 2004), barley stripe mosaic virus (BSMV) (Lim et al., 2008), potato virus X (PVX) (Schepetilnikov et al., 2005) and beet necrotic yellow vein virus (BNYVV) (Erhardt et al., 2000). Our transient expression assay found that the C-terminally tagged TGBp1-GFP (but not the N-terminally tagged GFP-TGBp1), expressed in the absence of other TGB proteins, co-localized with markers for both the PD and the ER in *N. benthamiana* leaves (Figures 1A,B, 2A). TGBp1 protein is not generally associated with PD when expressed alone. Expression of GFP-tagged TGBp1 proteins of BSMV and PMTV produced only diffuse fluorescence in the cytoplasm and nucleoplasm (Lawrence and Jackson, 2001; Zamyatnin et al., 2004), while BNYVV TGBp1 requires TGBp2 and TGBp3 proteins to target PD (Erhardt et al., 2000). The TGBp1 protein of CPMMV appears to be different in this respect.

TGBp2 is an integral membrane protein that is related to ER and ER-derived granular vesicles (Mitra et al., 2003; Haupt et al., 2005; Ju et al., 2005; Lim et al., 2009). The GFP-labeled TGBp2 protein of poa semilatent hordeivirus (PSLV) localizes to intracellular membrane systems such as the ER and Golgi apparatus (Solovyev et al., 2000; Zamyatnin et al., 2002), while the TGBp2 protein of grapevine rupestris stem pitting-associated virus (GRSPaV) is associated with the ER and ER-derived structures when expressed in BY-2 cells (Zamyatnin et al., 2002; Rebelo et al., 2008) and the TGBp2 of PVX, PMTV, BSMV, and other viruses have the properties of membrane proteins related to ER (Haupt et al., 2005; Ju et al., 2005; Lim et al., 2009). TGBp2 of CPMMV also localized to the ER (Figures 1D, 2C) but, unlike those encoded by most other viruses studied, also localized to PD (Figures 1C, 2B).

The strategy by which the three TGB proteins cooperate to enable viral movement has been well studied in some viruses (Morozov and Solovyev, 2003; Verchot-Lubicz et al., 2010). Among members of the genus *Potexvirus*, TGBp2 physically interacts with TGBp3 in a membrane-associated form and targets PD by two pathways: TGBp2-induced granular vesicles (carrying TGBp2 and TGBp3) and the sorting signals of TGBp3 (Schepetilnikov et al., 2005; Samuels et al., 2007). In addition, the TGBp2-TGBp3 complex also interacts with TGBp1, and assists the infectious complex (such as TGBp1-virions) in passing through PD (Erhardt et al., 2000; Zamyatnin et al., 2004; Lim et al., 2008). In BaMV, two conserved cysteine residues (Cys-109 and Cys-112) at the C-terminus of TGBp2 are critical for enhancing TGBp2- and TGBp3-dependent PD localization of TGBp1 (Ho et al., 2017). In this study of the carlavirus CPMMV, TGBp1, TGBp2, and TGBp3 interacted with each other as expected, and TGBp2 enhanced the interaction between TGBp1 and TGBp3 (Figures 3D,J). TGBp2 was targeted to PD, which is unusual among TGBp2 proteins but this could only be demonstrated when GFP was fused to its N-terminus. The C-terminal TM domain of TGBp2 was crucial for this targeting and its function may perhaps have been impaired if GFP was fused nearby. This C-terminal domain was also essential for the interaction between TGBp2 and either TGBp1 or TGBp3 (Figures 3, 4). Collectively, it is speculated that for CPMMV-encoded TGB proteins, TGBp2 protein physically interacts with TGBp3 and targets PD, while TGBp2 also recruits TGBp1 through the C-terminal TM domain, and so enhances the interaction between TGBp1 and TGBp3.

## Data Availability Statement

The original contributions presented in the study are included in the article/Supplementary Material, further inquiries can be directed to the corresponding author/s.

## Author Contributions

CJ, ZW, and ZS designed the research. CJ, SS, YH, HZ, CM, and YL performed the experiments. CJ drafted the manuscript. ZW and ZS revised the manuscript. ZW, ZS, and JC supervised the project. All authors read and approved the final manuscript.

## Conflict of Interest

The authors declare that the research was conducted in the absence of any commercial or financial relationships that could be construed as a potential conflict of interest.

## Publisher’s Note

All claims expressed in this article are solely those of the authors and do not necessarily represent those of their affiliated organizations, or those of the publisher, the editors and the reviewers. Any product that may be evaluated in this article, or claim that may be made by its manufacturer, is not guaranteed or endorsed by the publisher.

## Footnotes

1
http://www.enzim.hu/hmmtop/

2
https://tmdas.bioinfo.se/

3
http://split.pmfst.hr/split/4/
