# Supplementary material for: The C-Terminal Transmembrane Domain of Cowpea Mild Mottle Virus TGBp2 Is Critical for Plasmodesmata Localization and for Its Interaction With TGBp1 and TGBp3
Source: Front Microbiol. 2022 Apr 15;13:860695. doi: 10.3389/fmicb.2022.860695 (PMC9051516; doi:10.3389/fmicb.2022.860695)
Supplement: Supplementary file 1 [file Data_Sheet_1.pdf]

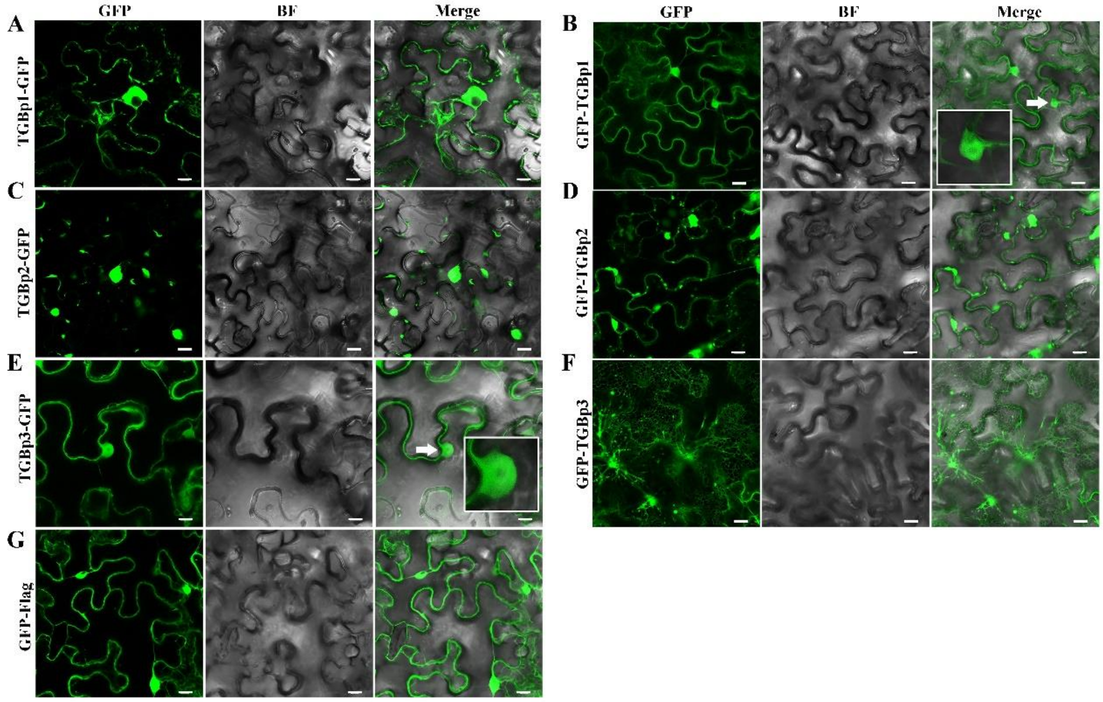

Supplementary Figure 1 Subcellular localization analysis of CPMMV TGBs protein in *N. benthamiana* leaf cells

Confocal images of *N. benthamiana* cells showing the subcellular localization patterns of TGBp1-GFP (A), GFP-TGBp1 (B), TGBp2-GFP (C), GFP-TGBp2 (D), TGBp3-GFP (E), GFP-TGBp3 (F) and GFP-Flag as the contorl (G). The place indicated by the white arrow is enlarged and displayed. Scale bars, 20 μm.

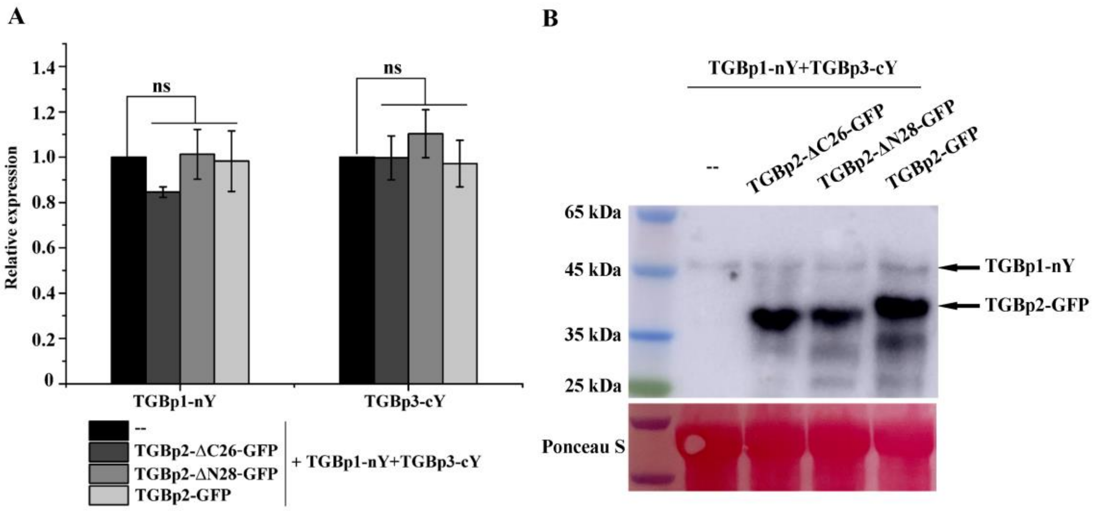

Supplementary Figure 2 Relative expression levels of TGBp1-nY and TGBp3-cY in BiFC assay.

(A) RNA transcript levels of TGBp1-nY and TGBp3-cY in TGBp1-nY + TGBp3-cY + TGBp2-ΔC26-GFP, TGBp1-nY + TGBp3-cY + TGBp2-ΔN28-GFP and TGBp1-nY + TGBp3-cY + TGBp2-GFP in BiFC assay using RT-qPCR, *N. benthamiana* leaves injected with TGBp1-nY+TGBp3-cY was used as a control, and the *N. benthamiana* UBC gene was used as an internal reference gene. (B) The protein expression levels of TGBp1-nY in BiFC assay as determined by anti-GFP antibody (HT801, TransGen).

Supplementary Table 1 Prediction of the transmembrane (TM) domains of the CPMMV TGBp2 protein by different methods

Algorithm	TM1 location (aa)	TM2 location (aa)
HMMTOP	12-29	77-93
DAS	15-31	75-92
SPLIT	18-37	75-97

Supplementary Table 2 Primers used in this study

Primer Name	Sequence
pCAMBIA-TGBp1-cY/Myc/GFP-F	5'-CGACGACAAGACCGTCACCATGAATGAACTGATCAGTAA-3'
pCAMBIA-TGBp1-cY/Myc/GFP-R	5'-GAGGAGAAGAGCCGTCGCTCAGAGTTTGGATAGGTTG-3'
pCAMBIA-TGBp2-nY/Flag/GFP-F	5'-CGACGACAAGACCGTCACCATGCCACTGACTCCAC-3'
pCAMBIA-TGBp2-nY/Flag/GFP-R	5'-GAGGAGAAGAGCCGTCGGTGAACCCTATTGCAG-3'
pCAMBIA-TGBp3-nY/cY/Myc/Flag/GFP-F	5'-CGACGACAAGACCGTCACCATGTCTGCAATAGGGTTCAC-3'
pCAMBIA-TGBp3-nY/cY/Myc/Flag/GFP-R	5'-GAGGAGAAGAGCCGTCGCAACCTACAA CTTAGTATCT-3'
pCAMBIA-TGBp2-ΔN28-GFP-F	5'-CGACGACAAGACCGTCACCATTAACAGATACGTTTATCC-3'
pCAMBIA-TGBp2-ΔC26-GFP-R	5'-GAGGAGAAGAGCCGTCGACCAAAGAGAATTTCAAGTT-3'
qPCR-TGBp1-F	5'-GACTGGGCAGCAAACTAGGG-3'
qPCR-TGBp1-R	5'-TTACAACTTTGCAGCGGGTC-3'
qPCR-TGBp3-F	5'-TGGGAGCTTCTTAACAGGAGT-3'
qPCR-TGBp3-R	5'-CTCCCTTTGCTCCCTTACCA-3'
qPCR-NbUBC-F	5'-TTTCGGTCCTGATGATACTCCC-3'
qPCR-NbUBC-R	5'-CACAGAGCAAAGACTGGATTGA-3'
pBin-GFP-TGBp1-F	5'-ACGAGCTGTACAAGGGTACCATGAATGAACTGATCAGTAA-3'
pBin-GFP-TGBp1-R	5'-GCGGACTCTAGTTCATCTAGATTACTCAGAGTTTGGATAGG-3'
pBin-GFP-TGBp2-F	5'-ACGAGCTGTACAAGGGTACCATGCCACTGACTCCACCACC-3'

pBin-GFP-TGBp2-R	5'-GCGGACTCTAGTTCATCTAGATTAGTGAACCCTATTGCAGA-3'
pBin-GFP-TGBp3-F	5'-ACGAGCTGTACAAGGGTACCATGTCTGCAATAGGGTTCAC-3'
pBin-GFP-TGBp3-R	5'-GCGGACTCTAGTTCATCTAGATTACAACCTACAACTTAGTA-3'
